# Infantile Epsilon-Sarcoglycan (SGCE) Myoclonus-Dystonia: Diagnostic Pitfalls and Poor Response to Pharmacologic Treatment

**DOI:** 10.7759/cureus.100342

**Published:** 2025-12-29

**Authors:** Rita Pissarra, Leonor Dias, Raquel Sousa, Cláudia Melo

**Affiliations:** 1 Pediatrics, Unidade Local de Saúde São João, Porto, PRT; 2 Department of Gynecology, Obstetrics, and Pediatrics, Faculty of Medicine, University of Porto, Porto, PRT; 3 Department of Neurology, Unidade Local de Saúde São João, Porto, PRT; 4 Department of Clinical Neurosciences and Mental Health, Faculty of Medicine, University of Porto, Porto, PRT; 5 Child Neurology Unit, Department of Pediatrics, Unidade Local de Saúde São João, Porto, PRT; 6 Center for Health Technology and Services Research (CINTESIS) and Health Research Network Associated Laboratory (RISE), University of Porto, Porto, PRT

**Keywords:** childhood, epsilon-sarcoglycan gene, infancy, myoclonus, myoclonus-dystonia syndrome, neuropsychiatric disorders

## Abstract

Myoclonus in infancy is often benign; however, persistent or progressive forms may indicate an underlying neurologic disease, including genetic movement disorders such as myoclonus-dystonia syndrome, a rare genetic condition most often associated with pathogenic variants in the Epsilon-Sarcoglycan (SGCE) gene. Early-onset cases are particularly challenging due to overlap with benign, epileptic, and metabolic conditions.

We report a six-year-old girl with myoclonus beginning at 12 months of age, involving her head, neck, and arms, and a delayed onset of mild dystonia at five years. Neurological examination, neuroimaging, electroencephalography, and metabolic investigations were unremarkable. Genetic testing identified a heterozygous pathogenic SGCE variant, confirming the diagnosis. Trials with zonisamide, carbamazepine, sodium valproate, and clonazepam were ineffective; however, motor and cognitive development remained within the average range. Behavioral and attentional difficulties emerged later in childhood.

This case highlights the diagnostic challenges of early-onset SGCE myoclonus-dystonia presenting as isolated myoclonus. Genetic testing was essential for diagnosis, pharmacologic response was limited, and functional outcome remained favorable. The later emergence of behavioral symptoms underscores the need for long-term multidisciplinary follow-up.

## Introduction

Myoclonus is characterized by brief, shock-like, involuntary movements caused by muscular contractions or inhibitions [1]. These movements are not rhythmic or suppressible and are often triggered or exacerbated by voluntary movement. Myoclonus can be benign and physiologic (e.g., neonatal sleep myoclonus, benign infantile myoclonus) or pathologic, associated with epilepsy, metabolic or structural brain disorders, infections, toxins, or genetic syndromes [1-3].

Transient and benign myoclonus is relatively common in otherwise healthy children in the first year of age. However, when myoclonus is persistent, progressive, or functionally impairing, it should prompt evaluation for an underlying neurologic condition. Importantly, myoclonus may appear in isolation or as part of a broader movement disorder, coexisting with dystonia, tremor, or other abnormal motor phenomena. Among these, Epsilon-Sarcoglycan (SGCE) myoclonus-dystonia is a well-recognized genetic syndrome [2-4].

SGCE myoclonus-dystonia, a rare inherited disorder, is characterized by the coexistence of myoclonic jerks and dystonia, due to variants in the SGCE gene, which encodes a protein component of the dystrophin-glycoprotein complex [5,6]. SGCE myoclonus-dystonia follows an autosomal dominant inheritance pattern with maternal imprinting, and its estimated prevalence is approximately 2 cases per 1,000,000 individuals in Europe, although the true prevalence may be underestimated given phenotypic variability and diagnostic challenges [7,8]. Symptoms usually begin in childhood or adolescence, often with spontaneous or action-induced myoclonus as the initial presentation. Timely recognition is essential, as early diagnosis can guide both management and genetic counseling [7-9].

This case is reported to highlight the diagnostic challenges of early-onset SGCE myoclonus-dystonia and to expand current understanding of its evolving clinical and behavioral spectrum in early childhood.

## Case presentation

A two-year-old girl was referred to the child neurology clinic in March 2022 due to abnormal involuntary movements. Perinatal history was unremarkable. Abnormal movements were first noticed at 12 months of age. They were sudden and transient, involving the neck and upper limbs, occurring during wakefulness, mainly during eating or playing. There was no loss of consciousness or additional abnormal movements. Psychomotor development was age-appropriate, with independent walking achieved at 18 months. She had a maternal cousin with a neuromuscular disorder and another maternal cousin with chronic motor tics disorder.

On neurological examination at the age of two, multifocal, spontaneous, myoclonus was noted predominantly involving the neck, shoulders and upper limbs. At that time, dystonia was not recognized, although retrospectively mild dystonic posture of the upper limbs is acknowledged. The remaining neurological examination was normal.

The child was admitted to the department for video-electroencephalogram (v-EEG) monitoring and further investigation. The v-EEG captured dozens of trunk, neck, and upper-limbs episodes, consistent with positive multifocal myoclonus (duration of 100-200ms), without electroencephalographic correlation. Brain magnetic resonance imaging was normal. Laboratory investigations, including thyroid function tests, thyroid antibodies, ammonia levels, ceruloplasmin and muscle enzymes, were within normal limits. Additional metabolic investigations, including plasma amino acids, urine organic acids, acylcarnitines, pyruvate and lactate, were also normal. A detailed summary of all laboratory and metabolic investigation is provided in Table 1.

**Table 1:** Summary of laboratory and metabolic investigations.

	Patient value	Unit	Reference range
Thyroid-stimulating hormone	1.37	uUI/mL	0.40-7.00
Free T4	1.05	ng/dL	1.04-1.60
Anti-thyroid peroxidase antibodies	<0.2	UI/mL	<4.1
Anti-thyroglobulin antibodies	1.1	UI/mL	<5.6
Ammonia	56	umol/L	18-72
Ceruloplasmin	18.8	mg/dL	21.7-43.3
Creatine kinase	83	U/L	10-149
Aspartate Aminotransferase	44	U/L	<36
Alanine Aminotransferase	15	U/L	<29
Lactate Dehydrogenase	282	U/L	<300
Plasma amino acids profile	All analytes within reference ranges	-	-
Urine organic acids profile	All analytes within reference ranges	-	-
Acylcarnitines profile	All analytes within reference ranges	-	-
Pyruvate	0.09	mmol/L	0.08-0.16
Lactate	0.6	mmol/L	0.5-1.6

Clinical exome sequencing revealed a pathogenic variant in heterozygosity in SGCE gene c.158C>G (p.Ser53*), consistent with SGCE myoclonus-dystonia syndrome. Parental testing showed that the mother had a normal SGCE genotype, while the father carried the same variant.

At age three, myoclonus persisted during action (less frequently in rest), particularly during tasks such as drawing and eating. Treatment with zonisamide was started without benefit, and switching to carbamazepine also resulted in no improvement.

Formal psychological evaluation was performed at the age of five, using the Wechsler Preschool and Primary Scale of Intelligence - Revised, and revealed a verbal intelligence quotient of 99 (47th percentile), a performance intelligence quotient of 92 (30th percentile) and a full-scale intelligence quotient of 95 (37th percentile), indicating average cognitive functioning. Preschool reports suggested good adaptation, with increased communication and interaction with peers and adults, as well as cooperation and interest in task completion.

At age five, parents reported emerging behavioral issues, describing the child as highly active and testing boundaries, while teachers noted distractibility in the kindergarten setting. On examination, attention was easily diverted. She presented upper limbs and cervical myoclonus during action and more rarely in rest. During drawing, laterocollis and contralateral upper limb mirror dystonia were observed, contributing to an apparent slowness in this task. During walking and running, some dystonic postures of one of the upper limbs were noticeable, as well as reduced unilateral arm swing. A therapeutic trial with valproate yielded no benefit, and a trial with clonazepam was associated with agitation and worsening of myoclonus, which led to discontinuation. The child was subsequently assessed and followed by psychology. Currently, parents report less frequent and more subtle myoclonus, without significant interference in daily activities. Symptoms exacerbate during intercurrent infections. The child continues to be regularly assessed by pediatric neurology, and it has been agreed with the parents that further therapeutic trials with other drugs will be attempted or other options considered if symptoms become functionally impairing.

Video 1 demonstrates myoclonus and dystonia recorded at different stages of follow-up, illustrating the progression and variability of motor symptoms over time.

**Video 1:** Epsilon-Sarcoglycan (SGCE) myoclonus-dystonia progression.

## Discussion

The diagnosis of SGCE myoclonus-dystonia in very young children is particularly challenging. When early manifestations are limited to isolated myoclonic jerks, the differential diagnosis includes benign myoclonus of infancy, epileptic or metabolic disorders, and secondary causes such as infections or toxins [4,9]. In such cases, detailed clinical observation, systematic exclusion of alternative etiologies, and ultimately genetic testing may be essential for diagnostic confirmation. In our patient, extensive laboratory, metabolic, and neurophysiologic investigations were unremarkable, effectively excluding secondary causes of myoclonus and supporting the pursuit of targeted genetic testing. The genetic result also underscores the relevance of maternal imprinting in SGCE-related disorders, as the pathogenic variant was inherited from an asymptomatic father. This finding has important implications for genetic counseling regarding recurrence risk in siblings and reproductive implications for the family.

What distinguishes this case from previously reported SGCE myoclonus-dystonia cases is the combination of an unusually early onset of myoclonus at 12 months of age, a prolonged period of isolated myoclonus without overt dystonia until five years of age, poor pharmacologic response, and the later emergence of behavioral and attentional difficulties despite preserved cognitive development. In addition, the identification of a pathogenic SGCE variant inherited from an asymptomatic father is consistent with the known imprinting mechanism and incomplete penetrance of this disorder. Together, these features expand the recognized phenotypic spectrum of SGCE myoclonus-dystonia in early childhood.

In this disorder, motor features often follow recognizable patterns. Myoclonus typically involves the upper limbs, trunk, and cervical region, and may appear with action or at rest. Dystonia, most often affecting the neck or limbs, may be subtle or absent at early stages [6,7]. Children may exhibit distinctive motor patterns, including “writer’s cramp” during writing or peculiar gait disturbances such as “circular shaking leg”, “dragging leg”, or “hobby-horse gait” in the early stages of the disease [9]. Our patient developed only mild dystonia after prolonged isolated myoclonus, delaying clinical suspicion. The onset at 12 months was unusually early compared with most reported cases, which typically arise in later childhood or adolescence [6-9].

Another important diagnostic consideration is the frequent association of SGCE myoclonus-dystonia with psychiatric comorbidities, including anxiety, depression, phobias, and obsessive-compulsive traits. These features may precede or accompany motor symptoms and are considered part of the disease spectrum [10]. In our patient, neuropsychiatric features were absent at initial assessment; however, by age five, emerging behavioral and attentional difficulties were noted, requiring psychological follow-up. This highlights the importance of early recognition of neuropsychiatric involvement, even in pediatric-onset cases.

Therapeutic management is often unsatisfactory. Antiseizure medications such as clonazepam and zonisamide, as well as anticholinergics and neuroleptics, may offer partial benefit [5,11,12]. Deep brain stimulation is reserved for severe or refractory cases [13]. In our patient, none of the medications (zonisamide, carbamazepine, or valproate) produced a significant benefit, reflecting the limited efficacy of pharmacologic therapy in early-onset SGCE-positive cases. Clonazepam was associated with agitation and paradoxical worsening of myoclonus, which led to discontinuation. Paradoxical responses to benzodiazepines such as this have been observed in young children and are thought to reflect the immaturity of inhibitory GABAergic circuits, resulting in cortical disinhibition and exacerbation of involuntary movements. [14] Alcohol responsiveness, a diagnostic clue often described in adolescents and adults, has no role in the pediatric setting.

Despite minimal treatment response, this patient currently demonstrates low functional disturbance, with apparent gradual reduction of the myoclonus severity, as well as maintenance of cognitive and motor development. Nevertheless, the later development of behavioral challenges suggests that psychiatric and neurodevelopmental features may evolve with age. Multidisciplinary follow-up is therefore essential to anticipate and manage both motor and non-motor complications. While long-term outcomes for SGCE myoclonus-dystonia are generally favorable, some pediatric-onset cases may follow a progressive or fluctuating course with significant early-life disability, reinforcing the need for ongoing monitoring and individualized interventions [10,15].

This case emphasizes the importance of considering SGCE myoclonus-dystonia in infants and toddlers presenting with unexplained, persistent myoclonus, even if the dystonia is not noticeable. Early targeted genetic testing can establish a definitive diagnosis, guide family counseling, and prevent unnecessary or potentially harmful interventions. Careful documentation of early-onset cases contributes to a better understanding of SGCE myoclonus-dystonia in pediatric populations and reinforces the value of vigilance in identifying movement disorders in early childhood.

## Conclusions

This case illustrates the complexity of diagnosing SGCE myoclonus-dystonia in very young children, particularly in the absence of dystonia or a contributory family history. Exome sequencing enabled early diagnosis and prevented unnecessary investigations.

Despite a limited pharmacological response, the child maintained favorable functional and cognitive outcomes. This case highlights the phenotypic variability of SGCE myoclonus-dystonia in childhood and underscores the importance of individualized management and long-term multidisciplinary follow-up, while recognizing the limitations of single-case observations.
